# Effects of a Mindfulness and Acceptance-Based Program on Intimate Relationships in a Youth Sample: A Randomized Controlled Trial

**DOI:** 10.3390/bs11060084

**Published:** 2021-06-09

**Authors:** María de Lourdes Rosales-Villacrés, Cristián Oyanadel, Diana Changotasig-Loja, Wenceslao Peñate-Castro

**Affiliations:** 1 Departamento de Psicología, Universidad de Concepción, Concepción 4070386, Chile; mariarosales@udec.cl (M.d.L.R.-V.); coyanadel@udec.cl (C.O.); 2 Facultad de Ciencias Psicológicas, Universidad Central del Ecuador, Quito 170129, Ecuador; dcchangotasig@uce.edu.ec; 3 Departamento Psicología Clínica, Psicobiología y Metodología, Facultad de Psicología y Logopedia, Universidad de La Laguna, Campus de Guajara, 38200 La Laguna, Spain

**Keywords:** intimate relationships, mindfulness-acceptance interventions, randomized controlled trial, youth

## Abstract

Intimate relationship conflicts in young people are crucial experiences for change. They can lead to more or less satisfactory relationships, depending on individuals’ skills to cope with these conflicts. This may or may not lead to violence in couples. Acceptance and self-regulation processes are an effective strategy to address individual factors such as avoidance and anxiety in intimate relationships of people in these age groups, thus preventing violence. The aim of this study was to assess the effects of an eight-session mindfulness and acceptance-based program (MAP). Participants (n = 40), who were aged from 18 to 25 years old, were randomly assigned to a group receiving the MAP or an active control group. Outcome measures were anxiety about abandonment, intimacy avoidance (Experiences in Close Relationships scale), well-being (Psychological Well-being Scale), dispositional mindfulness (Five Facet Mindfulness Questionnaire) and flexibility (Acceptance and Action Questionnaire-II). Measures were taken at pre-intervention, post-intervention and follow-up. Results showed that the MAP decreased anxiety (*p* = 0.025) and avoidance (*p* = 0.01) and increased mindfulness (*p* < 0.001) and flexibility (*p* = 0.001). In general, these improvements persisted at follow-up. Results are discussed in relation to the usefulness of mindfulness-acceptance strategies to cope with non-pathological intimate relationship conflicts.

## 1. Introduction

According to the World Health Organization (WHO), domestic violence often takes place within couples. It can take the form of physical, psychological, sexual or economic violence or of abuse, coercion, threats or control [1]. The WHO considers domestic violence as a public health problem with four factors—individual, relational, social and community-related—and recommends an ecological approach to address it [2]. According to this framework of reference, it is necessary to address individual and relational factors in order to influence the dynamics of violence, assuming that by doing so the other factors are redefined.

Intimate relationships go through several stages from casual and uncommitted dating until the creation of a relational bond with a greater involvement, intimacy and commitment [3]. However, it has been observed that higher degrees of seriousness and stability in a relationship are also associated with greater conflict [4]. This is because both members of the relationship must learn to know each other much better and to handle and solve disagreements [3]. This can lead to relationships of dominance as well as violent behaviors [5]. 

Conducting interventions among young people may be relevant considering that this population group must face major changes, including the search for stability and commitment in relationships. At this stage of the life cycle of individuals, intimate relationships are important for the consolidation of identity; this is because such relationships develop not only the responsibility and commitment of individuals but also their ability to love another person [4,6,7]. The stage between 18 and 29 years old is considered to be one of the critical periods of development, as it combines conflicts that are inherent to being in love, job instability and decision making about the future [6]. During this stage, intimate relationships are usually more committed and longer than at younger ages and may or may not include sexual intimacy, emotional closeness and the ability to care of each other, which are typical characteristics of adult relationships [8]. These characteristics could lead young people to learn more adaptive tools to cope with psychological distress and contribute to a more satisfactory relationship.

Emerging adulthood [9] is a very important stage to explore identity and achievement that differs from adolescence and full adulthood. Arnett has argued that the expression “young adults” may be improper because it conveys the idea that adulthood has been achieved. Yet, to the question “do you think you have reached adult life?”, most people aged 20–29 respond “in some respects yes, for others not”, showing that they are in a transition phase, where the transition to adulthood is close but not yet reached. In contrast, individuals over the age of 30 begin to perceive themselves more and more frequently as adults. On the basis of these considerations Arnett proposes to distinguish three phases: adolescence (10–18 years), emerging adulthood (19–29 years) and adult age (after 30 years). For this reason, emerging adults are in a privileged phase because they are not in their first romantic experiences and are in the transition phase that leads to the formation of a new family [10,11].

These processes in terms of commitment can be developed through acceptance and mindfulness-based interventions (MBIs). MBI is the term used for a series of programs, therapies and training initiatives that include mindfulness as one of their components [12]. They can be classified into two generations of programs [13]. The first generation is that of pioneer standardized mindfulness programs, Mindfulness-Based Stress Reduction (MBSR) [14] and Mindfulness-Based Cognitive Therapy (MBCT) [15], which have proven to be effective in promoting health and well-being in the general population and clinical populations. The second generation emerged in recent years and includes programs specifically focused on training in compassion, such as Compassion Focused Therapy (CFT) [16] and Mindful Self-Compassion (MSC) [17]. 

MBIs have emerged as therapeutical resources that can help individuals to raise their level of awareness of their relationships and increase emotional acceptance, promoting greater closeness [7,18,19,20]. There are several mindfulness-based programs that directly or indirectly promote well-being in intimate relationships [21,22,23,24]. 

Intimate relationships work better when each individual has personal balance and also finds balance in the relationship. The process of achieving personal balance implies regulating one’s own emotions and developing acceptance and compassion towards oneself and others. The process of achieving balance in a relationship implies building emotional presence and experiencing and expressing compassion for and acceptance of one’s partner [25]. These processes can be developed through mindfulness and Acceptance and Commitment Therapy (ACT).

Mindfulness is the practice of purposely focusing one’s attention on the present moment as it is without judgment or reaction, regardless of whether the experience is pleasant or not [14]. It teaches individuals to relate to negative feelings through processes and strategies such as observing, describing, acting with awareness, not judging and not reacting [26]. Moreover, it has been found that the lack of mindfulness and intolerance of uncertainty can predict problems such as anxiety and depression [27]. 

Mindfulness promotes non-judgmental attitudes, experiential awareness and acceptance, presence, openness and compassion towards oneself and others [28,29]. This makes it possible to transform relationships and experience greater satisfaction [30]. There are essential qualities of interpersonal attention that improve relationships. They are openness, empathy, compassion, kindness, joy at the well-being of others, enjoying the joy and happiness of others and equanimity. These qualities make individuals feel understood, accepted and loved, reducing their fears and defenses and leading to peaceful, empathetic and more connected relationships [31,32]. Another key element is self-compassion, which is linked to healthier elements in affective relationships, such as being more caring and providing support [17]. Self-compassion implies being kind and understanding with oneself when suffering instead of being excessively self-critical; perceiving one’s own experiences as part of human nature and keeping painful thoughts and feelings in one’s conscience so as not to overidentify with them [33].

In intimate relationships, mindfulness focuses on emotional self-regulation and self-acceptance processes to reduce reactivity at times of tension in the relationship. This can be achieved by being emotionally present, being compassionate and accepting the experience without judging [34].

ACT is an intervention for changing behaviors that has proven to be useful for individuals in their various problem areas [35]. It has two objectives: the first one is to lead clients to accept the aspects of their experience (e.g., thoughts, emotions, memories) that they have unsuccessfully been trying to change; the second one is to prevent those private events from paralyzing the lives of clients. Thus, ACT is not only aimed at reducing the symptoms in the life of clients. Therapy is structured on the basis of the elements of the Hexaflex model [36]. The Hexaflex model refers to the set of skills that contribute to psychological flexibility or inflexibility. According to the model, there are six dimensions of psychological flexibility (i.e., present moment awareness, acceptance, defusion, self-as-context, committed action, values). They are adaptive skills that enable individuals to experience uncomfortable thoughts, feelings and experiences in a flexible way. Practicing these skills routinely is considered to contribute to a fulfilling life. There are also six dimensions of psychological inflexibility (i.e., lack of contact with the present moment, experiential avoidance, self-as-content—a narrower vision of the self—cognitive fusion, lack of contact with values, inaction or rigid and reactionary behaviors). These are maladaptive reactions that promote psychological distress [37].

ACT, when applied to couples therapy, considers important elements of the psychological flexibility model such as the following [38]: experiential avoidance refers to how individuals try to manipulate the thoughts, emotions and feelings that emerge when interacting with their partner; creative despair identifies the problematic interaction pattern in the couple and analyzes its consequences in the short and long term; another element of the model is acceptance, not of the behavior itself but of the functions that it exerts by placing it in a different context that can be acceptable and less painful; finally, cognitive fusion occurs when the members of the couple over-identify with their thoughts about the present and the future and react to them as if they were literally true [39,40]. ACT develops the ability to distinguish the various roles played by the members of the couple in their lives, promoting the self as a context. It also makes it possible to identify the values of the relationship and consider them as directions chosen to make progress through commitment to action [39,40]. 

ACT-based interventions can be effective at enabling both members of the couple to be more flexible in their way of reacting both cognitively and emotionally during interactions; this makes it more likely that their choices in the relationship will be guided by their wishes and values [41].

Mindfulness is a resource that promotes individual self-regulation and influences wellbeing in intimate relationships. However, it reaches small effect sizes when used in isolation [19]. ACT promotes psychological flexibility, which not only enhances individual psychological well-being but is also associated with better functioning in intimate relationships [38]. We are not aware of any mindfulness-based programs that include the psychological flexibility proposal of ACT and are aimed at the individual well-being of people involved in a romantic relationship; in fact, most studies have been conducted with both members of the couple [42]. Integrating these two approaches is likely to lead to the construction of more mindful intimate relationships and prevent violent interactions.

This led to the Mindfulness and Acceptance-based Program (MAP), which combines the approaches of mindfulness and ACT in order to enable individuals to connect to themselves to generate psychological well-being and cultivate more mindful intimate relationships. The rationale is that the first relationship that needs to improve is the relationship with oneself (i.e., self-compassion). From that point, it is possible to build significant intimate relationships and prevent violent interactions.

The MAP is consistent with the recommendations of the WHO to reduce domestic violence. According to the ecological approach [1,2], variables affecting interpersonal relationships range from individual to community levels. Our objectives refer to an individual level of analysis.

The main aim of this study was to assess the effects of the MAP on the levels of intimacy avoidance and/or anxiety about abandonment experienced by young people in their intimate relationships.

## 2. Methods

### 2.1. Participants 

The study was conducted in the city of Quito, Ecuador. Participants were undergraduate university students. The sample was composed of 40 participants aged between 18 and 25 years, who were randomized into an active control group or an experimental group. The experimental group was composed of 8 men and 16 women (mean age = 22.5 years; SD = 1.91). The active control group included 6 men and 10 women (mean age = 22.4 years; SD = 1.93). The mean of relationship time was 22.8 months in the experimental group and 23.3 months in the control group.

Inclusion criteria were being aged between 18 and 25 years; being in an intimate relationship; not having any experience in mindfulness or meditation; and having high scores in avoidance/anxiety in the Experiences in Close Relationships scale. Exclusion criteria were being followed by a psychologist or psychiatrist for any mental health problems; having recently experienced any important losses; and having a physical condition that may influence the subject’s commitment to participate in the program. Although there were no restrictions and participation was individual, participants reported that there were 9 couples in the experimental group—8 heterosexual couples and 1 couple formed by two women.

The study was approved by the Ethics Committee of the Central University of Ecuador (code 0006-FCP-D-2017) and the trial is registered in the Cuban Public Registry of Clinical Trials (code RPCEC00000303).

### 2.2. Study Design 

We used a parallel randomized controlled trial. Participants were randomly assigned to an experimental group or an active control group. The experimental group received treatment in eight sessions conducted twice a week, with a duration of one month; the active control group received general information about relationships by email.

Participants were recruited by snowball sampling. People were invited to participate voluntarily by advertising the study in social media, visits to higher education institutions, posters and flyers.

Participants received the intervention material free of charge. It consisted of audio recordings to practice, a guidebook and a self-report sheet. At the end of follow-up, participants received a mindfulness card game. 

Participants were randomly assigned to the groups as soon as it was verified that they met the eligibility criteria. Simple randomization was used to generate the allocation sequence. An independent researcher not involved in the study established the groups, generated the randomization code and assigned the participants to the treatment groups following the established code. The same researcher also conducted the pre-post-follow-up assessments.

In this study we used a double-blind procedure: participants were not aware of the intervention they were assigned to (i.e., program or active control) and the independent researcher was also blinded to avoid any possible bias. Once the trial with the experimental group had ended, the real aim of the study was revealed to participants and the independent researcher.

### 2.3. Interventions

The MAP was designed to address individual and relational factors in order to build mindful relationships and prevent violent interactions. It combined mindfulness and ACT.

Interventions were conducted from September 2019 to March 2020, when the MAP was delivered to the active control group. 

The MAP undergone by the experimental group included eight group sessions with a duration of two hours each (see Appendix A, Table A1). Participant blocks were composed of an average of eight people, who attended two face-to-face sessions a week for one month. Each session had the same structure: it started with a mindfulness session and a reminder of the important elements of the previous session and of the weekly practice. This was followed by the central topic of the session in a process that included the following stages: experimentation, reflection, theory and practice in couple relationships. It ended with an explanation the exercise’s participants had to perform at home and an assessment of the level of wellbeing of participants at the end of the day (see Appendix B, Table A2). The MAP included exercises of mindfulness (e.g., conscious breathing, body scan, emotion meditation) and acceptance (e.g., functional analysis, DRAIN: disconnection, reactivity, avoidance, inside-your-mind, neglecting values; value identification).

The sessions were led by clinical psychologists trained to manage the program. We opened a call for clinical psychologists through the social media. After reviewing their background, the most qualified ones were interviewed. Those who had knowledge and skills regarding mindfulness considered important in the literature were invited to receive training [43]. After receiving theoretical and practical training on the program, facilitators underwent a theoretical test (i.e., a questionnaire assessing their knowledge) and a practical test (i.e., on how to conduct a session of the program). The four facilitators with the best results were selected. Each facilitator led one subgroup of the experimental group. To ensure an appropriate implementation of the program, the facilitator had to complete a self-report about compliance with the protocol in each session. After obtaining the consent of participants, two sessions were video recorded in each group and assessed with a checklist. The checklist was used to assess compliance with all the elements programmed for each session: the objective, the activities planned and the procedure followed. There was a section on observations to record any changes during the session. Facilitators were assigned to groups according to their availability. There were two groups in morning sessions and two groups in afternoon sessions.

The overall goal of the program was to connect with oneself in order to generate psychological well-being and cultivate intimate relationships with greater awareness. The sessions dealt with the following topics: wellbeing and mindful intimate relationships; myths about love; disconnection in intimate relationships; managing psychological distress in intimate relationships; healing past and/or significant relationships; acceptance: one day of silence; value-guided action; and full life and mindful intimate relationships.

The active control group was also organized in blocks of eight participants, who attended two face-to-face sessions: initial (i.e., pre) assessment and final (i.e., post) assessment. Participants were provided information about relationships in general in six emails and asked to complete a short, simple questionnaire about each text received. The facilitators of the active control group received the responses, analyzed them and sent individual feedback to each participant about each questionnaire.

After the end of the programs undergone by the experimental and active control groups, participants received reminder emails about the date of the follow-up assessment that included reading materials (for the experimental group) and two animated short films on relationships (for the active control group). Participants were summoned again three months later for the follow-up assessment. 

Participants in the active control group underwent the program after the end of the follow-up. 

### 2.4. Measures

We collected sociodemographic information about participants (i.e., sex, age, level of education) and the duration of their intimate relationship through a questionnaire.

**Five Facet Mindfulness Questionnaire [44]**. This is a self-administered questionnaire that measures the tendency to act with mindfulness. We used the Spanish adaptation to the Chilean population [45]. Its 39 items are assessed on a Likert scale from 1 to 5 (i.e., “never or very rarely true” to “very often or always true”). The lowest score is 39 points and the highest score is 195. Items are grouped into five factors: Observing, Describing, Acting with awareness, Non-judging and Non-reactivity. There is no adaptation to the Ecuadorian population, so we conducted a confirmatory factor analysis (CFA) with a similar sample to that of the study (n = 392). CFA fit indices were good: (X^2^ = 158.95; CFI = 0.97; TLI = 0.95; SRMR = 0.06; RMSEA = 0.05). We obtained the following Cronbach alpha values with this sample: observing = 0.71, describing = 0.82, acting with awareness = 0.81, non-judging = 0.88 and non-reactivity = 0.68.

**Experiences in Close Relationships (ECR) scale [46]**. This scale assesses two dimensions: avoidance of intimacy and anxiety about abandonment, with 18 items each. It is responded on a 7-point Likert scale (1 = strongly disagree, 4 = neither agree nor disagree, 7 = strongly agree). Total scores on each subscale are the sum of the scores divided by the number of items. Higher scores indicate higher levels of anxiety or avoidance. In the adaptation of the instrument to the Ecuadorian population (n = 392 emerging adults), the CFA showed a good fit of the model to the data (X^2^ = 152.019; CFI = 0.95; TLI = 0.93; SRMR = 0.07; RMSEA = 0.07). The dimensions had good reliability in our sample: avoidance (α = 0.88) and anxiety (α = 0.89).

**Psychological Well-being Scale (PWS) [47]**. The scale assesses positive attributes of psychological wellbeing. We used the Spanish adaptation of the 39-item scale [48], which was shorter and included 29-items [49]. The PWS is responded on a Likert scale from 1 to 6 (i.e., “strongly disagree” to “strongly agree”). The scale is made up of six subscales: Self-acceptance, Positive relations with others, Autonomy, Environmental mastery, Personal growth and Purpose in life. Scores on the subscales are combined to obtain a total score of psychological wellbeing. There is no adaptation to the Ecuadorian population, so we conducted a CFA with a sample of 392 emerging adults. CFA values indicated a moderately acceptable fit of the model (X^2^ = 115.581; CFI = 0.90; TLI = 0.89; SRMR = 0.07; RMSEA = 0.08). The total alpha value of the instrument was 0.91. We obtained the following Cronbach alpha values with this sample: self-acceptance = 0.80, positive relations with others = 0.66, autonomy = 0.62, environmental mastery = 0.55, personal growth = 0.72, purpose in life = 0.81. The environmental mastery and autonomy dimensions showed low reliability.

Acceptance and Action Questionnaire II (AAQ-II) [50]. This instrument measures the degree of experiential avoidance/psychological flexibility. We used the version translated and adapted to the context of Ecuador [51]. It is composed of 10 items that are responded on a Likert scale with seven response options (from “never” to “always”) and has a one-factor structure. In this study, the Cronbach alpha internal consistency coefficient was 0.84. 

### 2.5. Data Analysis 

In a first analysis, we compared the baseline features of both groups with Student’s *t*-tests. To assess the effectiveness of the program, we applied a mixed design ANOVA with two factors. A between-subject factor was treatment, distinguishing between two groups: the control group, which received psychoeducation, and the experimental group, which underwent the program. A second factor was time, which was measured within subjects at three points in time: before participating in the program (i.e., pre), upon completion of the program (i.e., post) and at follow-up (i.e., fol). The variables we intended to explore referred both to the positive dimension (i.e., well-being/mindfulness) and the negative dimension (i.e., anxiety, avoidance). Thus, we finally obtained a mixed-design ANOVA model for each variable. Moreover, in the models for the avoidance and anxiety variables, we included “baseline level of avoidance” and “baseline level of anxiety” in each as a control variable for each subject, respectively. The levels of these variables were categorized for each subject before the experiment. Specifically, we measured anxiety and avoidance levels of each individual and classified them into 4 levels (i.e., low, moderate, high and very high). To assess whether the program had an influence or a significant effect on the variables of subjects, we assessed the interaction between the factors (i.e., time and treatment) with the F-test. If it was significant, the influence of each factor on the dependent variable was assessed with multiple comparisons of estimated means using the EMMeans model. Specifically, we verified whether there were or not differences between two treatment levels in each factor for each fixed-level value of the other factor (e.g., we assessed the existence or not of significant differences between the control and experimental groups at instant 1 at a fixed level of the time factor = 1 and determined whether there were differences or not between the control and experimental groups, that is, an influence of the treatment factor). This was done with multiple comparisons using post-hoc *t*-tests with the Sidak correction. If the interaction was not significant, we assessed the main effects of each factor separately. If the time factor was significant, we explored its influence with a repeated-measures ANOVA considering the three instants by means of the F-test. If the treatment factor was significant, we compared both groups with a *t*-test. SPSS version 22 software was used.

## 3. Results

We compared the baseline features of both groups with Student’s *t*-tests: Well-being (t = −0.576; *p* = 0.568), Mindfulness (t = 0.768; *p* = 0.446), Psychological flexibility (t = −0.450; *p* = 0.655).

### 3.1. Descriptive Parameters

Table 1 shows the mean and standard deviations of the control and experimental groups at pre, post, and follow-up for the variables Well-being, Psychological flexibility, Mindfulness, Anxiety and Avoidance.

### 3.2. Anxiety

**Figure 1:**
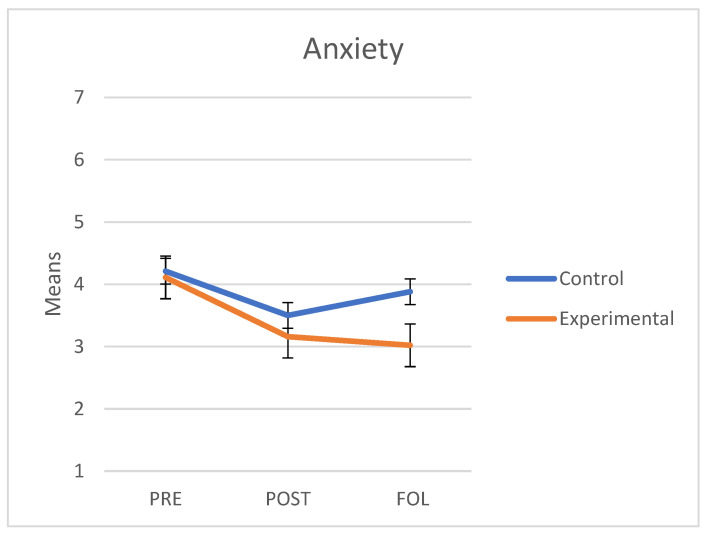
Mean scores in Anxiety about abandonment of the control and experimental groups at the three points in time (PRE: pre-treatment, POST: post-treatment, FOL: follow-up).

As regards anxiety, we verified that the assumptions of sphericity (*W* = 0.948; *p* = 0.379) and homogeneity of variance-covariance matrices were met with Box’s M test (F = 1.35; *p* = 0.229). The interaction between group and measures taken at the three points in time was significant with a medium effect size (F(2.74) = 3.88, *p* = 0.02, *η^2^* = 0.095). The main effect of group did not show any significant differences (F(1.37) = 0.99, *p* = 0.326, *η^2^* = 0.026). The main effect of time was not significant either (F(2.74) = 0.69, *p* = 0.505, *η^2^* = 0.018). As shown on Figure 1, both groups had similar mean scores at baseline (i.e., pre scores) and the scores of both groups decreased in the post measurement. At follow-up, however, anxiety continued to decrease in the experimental group but increased in the control group.

A separate analysis of each group revealed that in the control group there were significant differences between pre and post measures (t(45) = −2.75, *p* = 0.028) but not between pre and follow-up measures (t(45) = 1.45, *p* = 0.397) nor between post and follow-up measures (t(45) = 1.75, *p* = 0.243). By contrast, in the experimental group, we observed significant differences between pre and post measures (t(69) = −4.77, *p* < 0.001) and between pre and follow-up measures (t(69) = −6.56, *p* < 0.001); however, no differences were observed between post and follow-up measures (t(69) = −0.75, *p* = 0.842).

### 3.3. Avoidance

**Figure 2:**
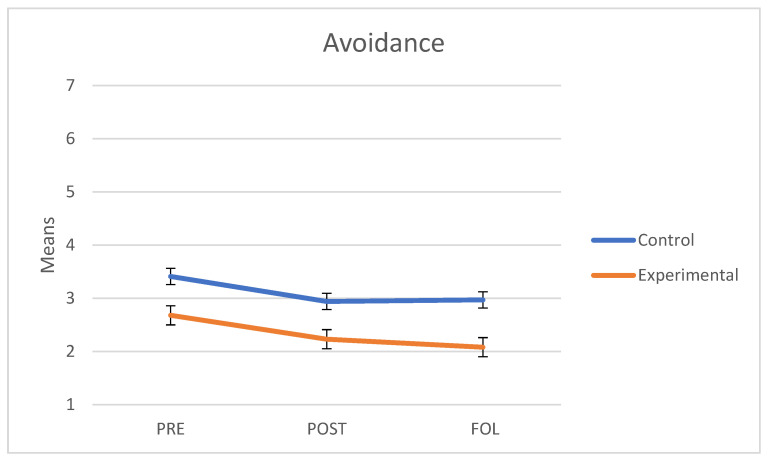
Mean scores in Avoidance of intimacy of the control and experimental groups at the three points in time (PRE: pre-treatment, POST: post-treatment, FOL: follow-up).

As regards avoidance, we verified that the assumptions of sphericity (*W* = 0.983; *p* = 0.734) and homogeneity of variance-covariance matrices were met with Box’s M test (F = 1.627; *p* = 0.135). The interaction between time and follow-up was not significant (F(2.74) = 2.46, *p* = 0.093, *η*^2^ = 0.062). The main effect of group was statistically significant in favor of the group that underwent the intervention program (i.e., lower avoidance) (F(1.37) = 8.51, *p* = 0.006, *η*^2^ = 0.187). The main effect of time was also significant (F(2.74) = 7.19, *p* = 0.001, *η*^2^ = 0.163). In fact, we observed a decrease in avoidance both at post-treatment and follow-up in both groups. Figure 2 shows a graphical representation of these results.

To explore which periods showed significant differences, we conducted post-hoc *t*-tests with the Sidak correction. Overall, the level of avoidance showed significant differences between pre and post measures (t(117) = −2.89, *p* = 0.019) and pre and follow-up measures (t(117) = −3.42, *p* = 0.004); by contrast, no significant changes were found between post and follow-up measures (t(117) = −0.63, *p* = 0.897), in which the means showed a progressive decrease. This shows that the level of avoidance dropped significantly from the pre to the post measure and remained stable from then onwards.

After determining the effects of the program on the two main outcome variables, we explored its influence on the measures of mindfulness, flexibility and wellbeing and the potential changes it may have led to. 

### 3.4. Mindfulness

**Figure 3:**
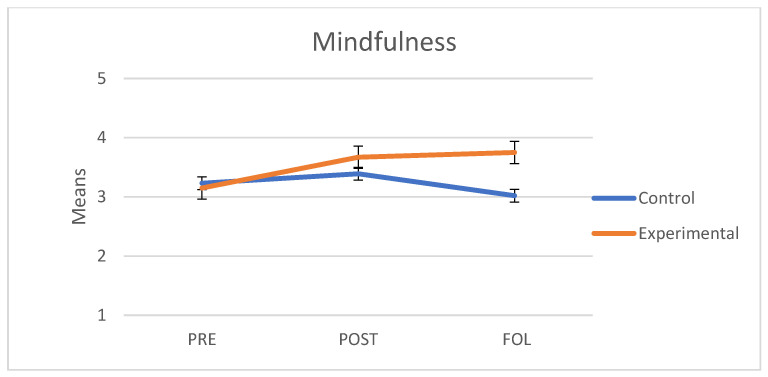
Mean scores in Mindfulness of the control and experimental groups at the three points in time (PRE: pre-treatment, POST: post-treatment, FOL: follow-up).

As regards mindfulness (Five Facet Mindfulness Questionnaire), we verified that the assumptions of sphericity (*W* = 0.866; *p* = 0.07) and homogeneity of variance-covariance matrices were met with Box’s M test (F = 2.039; *p* = 0.057). The interaction between group and the three points in time was significant and had a large effect size (F(2.76) = 14.22, *p* < 0.001, *η*^2^ = 0.272). The main effect of group showed significant differences (F(1.38) = 4.84, *p* = 0.034, *η*^2^ = 0.113). The main effect of time was also significant (F(2.76) = 7.05, *p* = 0.005, *η*^2^ = 0.157). As shown on Figure 3, both groups had similar mean scores at baseline (i.e., pre scores). In the experimental group, mindfulness scores had increased at the end of the program and continued to increase at follow-up; in the control group, however, the levels of mindfulness slightly improved in the post measure but decreased at follow-up to levels even lower than those of the pre measure.

A separate analysis of each group revealed that in the control group there were no significant differences between pre and post measures (t(45) = 0.98, *p* = 0.699) nor between pre and follow-up measures (t(45) = −1.85, *p* = 0.199). However, we observed significant differences between post and follow-up measures (t(45) = −3.41, *p* = 0.004). By contrast, in the experimental group, we observed significant differences between pre and post measures (t(69) = 5.32, *p* < 0.001) and between pre and follow-up measures (t(69) = 4.91, *p* < 0.001); however, no differences were observed between post and follow-up measures (t(69) = 1.02, *p* = 0.671).

### 3.5. Experiential Avoidance/Psychological Flexibility

**Figure 4:**
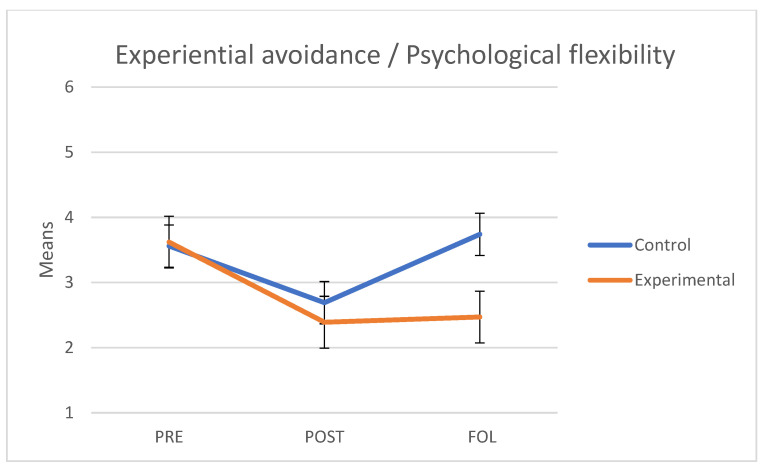
Mean scores in Experiential avoidance/Psychological flexibility of the control and experimental groups at the three points in time (PRE: pre-treatment, POST: post-treatment, FOL: follow-up).

As regards experiential avoidance/psychological flexibility (Acceptance and Action Questionnaire II), we verified that the assumptions of sphericity (*W* = 0.951; *p* = 0.397) and homogeneity of variance-covariance matrices were met with Box’s M test (F = 0.403; *p* = 0.878). The interaction between group and the three points in time obtained was significant and had a large effect size (F(2.76) = 7.54, *p* = 0.01, *η*^2^ = 0.166). The main effect of group showed non-significant differences (F(1.38) = 2.84, *p* = 0.10, *η*^2^ = 0.07). Yet, the main effect of time was significant (F(2.76) = 15.39, *p* < 0.001, *η*^2^ = 0.288. As shown on Figure 4, both groups had similar mean scores at baseline (i.e., pre scores); the scores of the experimental group were maintained at follow-up but the experiential avoidance scores of the control group increased.

A separate analysis of each group revealed that in the control group there were significant differences between pre and post measures (t(45) = −2.86, *p* = 0.021) but there were no significant differences between pre and follow-up measures (t(45) = 0.81, *p* = 0.810). We observed significant differences between post and follow-up measures (t(45) = 4.10, *p* = 0.001). By contrast, in the experimental group, we observed significant differences between pre and post measures (t(69) = −5.33, *p* < 0.001) and between pre and follow-up measures (t(69) = −4.48, *p* < 0.001); however, no differences were observed between post and follow-up measures (t(69) = 0.42, *p* = 0.967).

### 3.6. Psychological Well-Being

**Figure 5:**
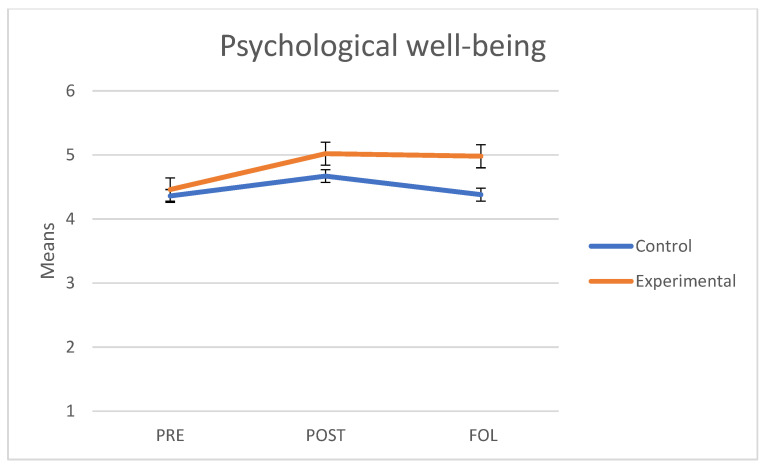
Mean scores in Psychological well-being of the control and experimental groups at the three points in time (PRE: pre-treatment, POST: post-treatment, FOL: follow-up).

As regards psychological wellbeing (Ryff’s Scales of Psychological Well-being), we verified that the assumptions of sphericity (*W* = 0.904; *p* = 0.155) and homogeneity of variance-covariance matrices were met with Box’s M test (F = 1.573; *p* = 0.150). The interaction between group and the three points in time was significant and had a medium effect size (F(2.76) = 5.52, *p* = 0.006, *η*^2^ = 0.127). The main effect of group did not show any significant differences (F(1.38) = 2.74, *p* = 0.106, *η*^2^ = 0.67). However, the main effect of time was significant (F(2.76) = 13.59, *p* < 0.001, *η*^2^ = 0.263). As shown on Figure 5, both groups had similar scores at baseline and higher scores at the end of the program. Yet, although the levels of the experimental group persisted at follow-up, those of the control group significantly decreased.

A separate analysis of each group revealed that in the control group there were no significant differences between pre and post measures (t(45) = 2.13, *p* = 0.114) nor between pre and follow-up measures (t(45) = −0.071, *p* = 1.000). Yet, we observed significant differences between post and follow-up measures (t(45) = −2.70, *p* = 0.030). By contrast, in the experimental group, we observed significant differences between pre and post measures (t(69) = 5.30, *p* < 0.001) and between pre and follow-up measures (t(69) = 4.64, *p* < 0.001); however, no differences were observed between post and follow-up measures (t(69) = −0.379, *p* = 0.975).

## 4. Discussion

According to the WHO, addressing domestic violence requires considering the individual, relational, social and community-related factors that maintain this violent interaction. The aim of this study was to assess the effects of the MAP on the levels of intimacy avoidance and anxiety about abandonment of young people in their intimate relationships. The intervention program applied (i.e., MAP) was based on the tenets of mindfulness meditation and the acceptance processes specially developed by ACT. In fact, our results seem to favor this combination as participants showed improvements in their emotional regulation strategies (regarding anxiety and avoidance) and in their flexibility in intimate relationships.

As regards the primary variables, results showed a decrease in anxiety levels between pre and follow-up measures in the experimental group; by contrast, no significant differences were found in anxiety levels in the active control group. As regards intimacy avoidance, both the experimental and active control groups showed a significant decrease in this variable between pre and post measures, and the decrease remained until follow-up. This decrease in anxiety and avoidance was corroborated by an increase in psychological well-being in the experimental group when we compared baseline levels with levels reached at follow-up. To verify internal validity, we expected the mindfulness and acceptance-based program to improve dispositional mindfulness and flexibility. This was confirmed by comparing pre and follow-up measures: subjects who underwent the MAP experienced an increase in their levels of mindfulness and psychological flexibility.

Analyzing the results for all variables, we observed that the active control group improved at post-treatment. This suggests that the contents provided to the active control group were also able to improve both avoidance and anxiety (as well as dispositional mindfulness, flexibility and wellbeing). Some examples of the contents were “Why is your brain always looking for trouble?”; “Empathy: how it can improve your life”; “Assertiveness: extending self-esteem to social relationships”. However, these improvements did not persist over time in the active control group but persisted in the experimental group. This result is consistent with the findings of another study that also used an active control group and whose effects were evidenced at follow-up [52]. The exception was intimacy avoidance, in which the improvements persisted at follow-up. A reason for this may be that the content of the materials provided to active control group participants may have had a direct influence on avoidance. In any case, the improvements of the experimental group were significantly greater. 

These data are also consistent with the findings on the practice of mindfulness and its effect on the well-being of individuals, particularly young people. In fact, the practice of mindfulness has been associated with positive variables for mental health (i.e., flexibility, resilience, good self-esteem), which in turn are associated with greater life satisfaction [53]. The findings on the effects of the MAP model on intimate relationships are consistent with those of other studies that have shown that psychological flexibility significantly increases well-being, life satisfaction and mindfulness skills [54]; they also agree with the results of studies that have shown that higher levels of inflexibility are associated with lower satisfaction of both members of the relationship, lower sexual satisfaction and emotional support, and higher levels of conflict such as abuse, avoidance and anxiety [38].

The improvements in psychological well-being of emerging adults in their intimate relationships can be understood from the practice of mindfulness as one of the most direct ways to improve the ability to self-soothe [25]. Thus, to approach psychological distress associated with intimate relationships it is necessary to conduct further research and consider attachment theory in greater detail as a dynamic and complex process [55].

## 5. Limitations

Given that our program intervention referred to an individual level of analysis, in accordance with the ecological approach advocated by the WHO, the extent of our results was limited because there are several other levels/systems that affect intimate relationships [2]. To fully understand such relationships, it is necessary to take into account the various systems that affect intimate relationships (i.e., relational, organizational, and community levels).

A critical element was the high dropout rate at the beginning of the program, which led to sample loss. This difficulty ensuring adherence to the program does not seem to be unique to our study. We found similar limitations due to group randomization in experimental studies on intimate relationships [52]. However, despite this, the data obtained were consistent, with medium and large effect sizes. The scarcity of studies with programs combining mindfulness and ACT makes it difficult to thoroughly compare our results on the effect on intimate relationships with those of other studies. However, our intention with this study was to open the door to new studies that may provide greater insight on this subject, which raises so much interest, and help to improve relationships.

The greatest limitation was the loss of sample size, which distorted the initial balance between the control and experimental groups in the variables anxiety and intimacy avoidance; as a result, both groups had different levels in avoidance at baseline. This caused a second limitation, which was the final sample size. In addition, we worked in isolation with only one of the partners in the relationship, but it would be more appropriate to apply the program to both partners. 

Other limitation of this study is that it was not possible to compare results between sexes because sample size was not large enough and the results would have been highly unstable. Another limitation of the study was that the final level of satisfaction with the program was not assessed. This would have provided valuable information. 

## 6. Future Lines of Research

In the future, it is necessary to complement this intervention (MAP) with others that consider social and community factors involved in domestic violence. It would be useful to test the program with couples of other ages and to differentiate between the results of men and women in this type of intervention. 

Moreover, it would be useful to experimentally assess the effectiveness of mindfulness and ACT approaches separately. Furthermore, it would be necessary to explore whether factors such as the length of the intervention and an online and self-reported format are effective or not at improving the quality of intimate relationships [52]; this would help to determine whether such relationships can be improved or not considering the ACT psychological flexibility model.

It would be interesting to conduct factorial randomized controlled trials to compare our program (MAP) to others; it would also be a good idea to include other instruments to measure relational mindfulness and assess its effect on significant relationships and not only intimate ones [56]. This would make it possible to identify those that are most useful to improve psychological wellbeing and the experience in intimate relationships of various populations such as those with chronic diseases [57] or physical or mental health problems affecting quality of life and of relationships. 

## 7. Conclusions

This is a pioneering study whose results show that combining mindfulness and ACT approaches is effective at modulating the levels of avoidance and anxiety in intimate relationships and increasing psychological well-being. These results are encouraging as they show the possibility of building healthy intimate relationships with greater emotional involvement and commitment.

## 8. Patents

Trial registration code: RPCEC00000303. Date of registration: 13-11-2019.

## Figures and Tables

**Table 1:** Mean scores and standard deviations of the experimental group (*n* = 24) and control group (*n* = 16) at pre, post and follow-up for the outcome variables.

	PRE				POST				Follow-Up			
Groups	EG		CG		EG		CG		EG		CG	
Variables	M	SD	M	SD	M	SD	M	SD	M	SD	M	SD
Well-being	4.46	0.64	4.36	0.65	5.02	0.7	4.67	0.67	4.98	0.65	4.38	0.77
Psychological flexibility	3.62	1.16	3.56	1.28	2.39	0.96	2.69	1.01	2.47	0.9	3.74	1.24
Mindfulness	3.15	0.33	3.23	0.4	3.67	0.53	3.39	0.55	3.75	0.55	3.02	0.64
Anxiety	4.11	1.29	4.21	0.99	3.16	1.25	3.5	1.2	3.02	1.16	3.88	1.04
Avoidance	2.68	1.04	3.41	0.84	2.23	0.85	2.94	0.71	2.08	0.71	2.97	0.97

Note: PRE = initial measurement, POST = measurement after treatment, EG = experimental group, CG = control group.

## Data Availability

Rosales, Lourdes (2021), “ECA-Mindfulness, ACT and relationship”, Mendeley Data, V1, doi:10.17632/9fb8vrj34c.1.

## Appendix A

**Table A1:** Comparison between interventions and contents in the experimental group and the active control group (2.3 Interventions).

Comparison between Program Contents				
	Experimental Group		Active Control Group	
Session No.	Title	Objective	Topics	
**1**	**Well-being and mindful intimate relationships**	Identify the gap that exists between idealization and the everyday reality of intimate relationships. Understand basic concepts about mindfulness, its effects and its practice. Pre-treatment assessment.	**Face-to-face session 1**	Pre-treatment assessment and explanation of the procedure to follow.
**2**	**Myths about love**	Identify the myths that exist about intimate relationships.	**Online psychoeducation, reflection and feedback**	**Text 1 for reading and reflection:** Why is your brain always looking for trouble?
**3**	**Disconnection in intimate relationships**	Understand how disconnection occurs in intimate relationships.		**Text 2 for reading and reflection:** Empathy: how it can change your life.
**4**	**Managing psychological distress in intimate relationships**	Acquire tools to manage psychological distress.		**Text 3 for reading and reflection:** Assertiveness: extending self-esteem to social relationships. A distinction is made between assertive, passive and aggressive individuals.
**5**	**Healing past and/or significant relationships**	Identify, accept and heal the wounds from past and/or significant relationships through compassion and self-compassion.		**Text 4 for reading and reflection:** Anger, sadness and pain: what is going on with today’s music?
**6**	**Acceptance: one day of silence**	Consolidate the practice of mindfulness to develop acceptance and psychological well-being through group sessions.		**Text 5 for reading and reflection:** Me! Me! Me! The Age of Digital Narcissism.
**7**	**Value-guided action**	Recognize the importance of values as a guide to action in life and intimate relationships.		**Text 6 for reading and reflection:** Loneliness is not being alone, but being empty, according to Seneca.
**8**	**Full life and mindful intimate relationships**	Design a plan to continue developing mindfulness, generate personal well-being and cultivate intimate relationships. Post-treatment assessment.	**Face-to-face session 2**	Post-treatment assessment.
**Reminder of the follow-up assessment (experimental group)**		Sending of reading materials.	**Reminder of the follow-up assessment (active control group)**	**Sending of animated short films on relationships:** This is the Plan The Spider and The Butterfly

## Appendix B

**Table A2:** Program “Mindfulness and development of well-being in intimate relationships between young people”. Session 1.

Title:	Well-Being and Mindful Intimate Relationships		
**Objectives:**	**1.** **Identify the gap that exists between idealization and the everyday reality of intimate relationships.** **2.** **Understand basic concepts about mindfulness, its effects and its practice.**		
**Activities**	**Process**	**Resources**	**Time**
Establishing a framework for the session.	Greetings and welcome. Record of attendance, email address and telephone number. Hand over the printed material and a folder. Set group rules. Individual presentation and expectations of participants. Explanation of how the sessions are conducted.	Presentation of the work space. Rules. Attendance record. Folders and printed sheets.	20′
Defining a baseline.	Application of tests or instruments, first measurement.	Record of sociodemographic data. Informed consent to participate in the program. Printed sheets with the instruments.	30′
Psychoeducation: Complexity in intimate relationships.	Build a group space. Create three groups. Distribute the puzzles. Instructions: “Put the puzzle together” After finishing the assignment, reflect upon the activity and its similarity with intimate relationships.	Puzzle Slate Markers	30′
Introduction to the program.	Think about the Fly meditation video. Presentation of basic concepts about mindfulness and its effects. Stages of stress: stimulus, resistance, exhaustion, wear in the intimate relationship. Build a space of freedom of choice instead of reaction: the triangle of attention and the basics of practice.	The Fly meditation video. https://www.youtube.com/watch?v=t7xqGvtHxew (accessed on 16 September 2019) Power point presentation.	20′
Breath-focused meditation.	Mindfulness exercise: feeling and breathing.	Audio recording, script, instructions for practice.	15′
Explanation of the exercises for the week.	**Formal practice:** Breath-focused meditation (audio recording) for 15 min. **Informal practice**: Place 5 color tags in five places you often visit in your everyday life, stop and take 3 mindful breaths feeling how your stomach expands and contracts. **Mindfulness:** Moments of waiting: observing thoughts, emotions and feelings.	Sheet. Audio recording for breathing. Color tags.	5′
Closure:	In few words: What am I taking home from this workshop? Assessment of well-being from 1 to 10. Create WhatsApp group for notifications and follow-up of practice. **Materials for next session**: Bring an extra pair socks.	Briefcase.	10′
